# Knowledge, attitude, and practice of osteoporosis prevention in elder patients following lumbar fusion surgery: a cross‐sectional study

**DOI:** 10.3389/fragi.2025.1543839

**Published:** 2025-06-10

**Authors:** Shaoying Sheng, Manman Wang, Chengfei Yu, Qing Li, Zhan Wang, Yaojing Ma

**Affiliations:** ^1^ Department of Nursing, The Second Affiliated Hospital of Zhejiang University School of Medicine, Hangzhou, China; ^2^ Department of Orthopedic Surgery, The Second Affiliated Hospital, Zhejiang University School of Medicine, Hangzhou, China; ^3^ Orthopedics Research Institute of Zhejiang University, Hangzhou, China; ^4^ Key Laboratory of Motor System Disease Research and Precision Therapy of Zhejiang Province, Hangzhou, China; ^5^ Zhejiang Provincial Clinical Medical Research Center for Motor System Diseases, Hangzhou, China

**Keywords:** osteoporosis, older adult female patients, lumbar fusion surgery, osteoporosis prevention and awareness tool (OPAAT), education programs

## Abstract

**Purpose:**

To assess the knowledge, attitudes, and practices (KAP) regarding osteoporosis prevention among older adult patients following lumbar fusion surgery and identify associated factors.

**Patients and methods:**

A cross-sectional study was conducted with participants aged ≥50 years. Data on demographic characteristics, KAP related to osteoporosis prevention, and lifestyle behaviors were collected using structured questionnaires. Chi-square tests were performed to explore associations between demographic variables and knowledge levels.

**Results:**

A total of 217 participants were included in the study. Participants demonstrated varying levels of knowledge, with 28.6% scoring high on the OPAAT. Female gender, higher educational attainment, urban residence, and diabetes were significantly associated with higher knowledge levels. Positive lifestyle practices, such as calcium and vitamin D supplementation, were observed in 64.5% and 41.0% of participants, respectively, while regular outdoor exercise and dairy product consumption were less common.

**Conclusion:**

The findings highlight gaps in osteoporosis prevention KAP among older adult patients following lumbar fusion surgery. Tailored educational programs and interventions are necessary to enhance awareness and promote healthier behaviors.

## Introduction

Osteoporosis is a prevalent condition among older adults, characterized by reduced bone density and increased fracture risk (Cosman et al., 2024; Quek et al., 2024; Wang et al., 2024). Notably, many patients remain untreated for osteoporosis before undergoing lumbar fusion surgery, particularly men. Untreated patients typically exhibit lower bone mineral density (BMD) and elevated bone resorption markers compared to those receiving treatment (Köhli et al., 2025). Furthermore, osteoporosis is a significant risk factor for mechanical complications of lumbar fusion (Filley et al., 2024) and anti-osteoporosis therapy could reduce bone resorption and thus facilitate fusion (S et al., 2024). Thus, adequate knowledge, positive attitudes, and appropriate practices (KAP) regarding osteoporosis prevention are essential to mitigating these risks. However, studies on osteoporosis-related KAP in this specific patient population are limited.

Postoperative recovery following lumbar fusion surgery often involves prolonged periods of reduced activity (Devin and McGirt, 2015; Wang et al., 2020), which can further compromise bone health. Additionally, the aging process itself, along with common comorbidities such as diabetes and cardiovascular disease, may exacerbate the risk of osteoporosis (Ross et al., 2022; Azeez, 2023). Despite the critical importance of osteoporosis prevention, there is a lack of targeted educational programs addressing this issue in the context of surgical recovery. Investigating the knowledge gaps and lifestyle behaviors of these patients can help identify specific barriers to effective osteoporosis prevention and guide the development of tailored interventions.

This study aims to evaluate the levels of knowledge, attitudes, and practices concerning osteoporosis prevention among older adult patients following lumbar fusion surgery. The findings can inform the development of targeted educational strategies and interventions to promote bone health in this high-risk group.

## Materials and methods

### Study participants

A cross-sectional study was conducted on older adult patients (aged ≥50 years) who underwent lumbar fusion surgery from April 2024 to July 2024. Participants were recruited from a tertiary hospital. Only patients with no previous diagnosis of osteoporosis were included in the study.

### Ethics statement

This study was reviewed and approved by the local Ethics Committee (IRB-2024–1492). Written consent was obtained from all participants. Informed consent was submitted by all subjects when they were enrolled.

### Sampling method

This study utilized a convenience sampling method, selecting eligible patients consecutively from those admitted for spinal surgery during the study period. While this approach enables efficient data collection within a specific population, it may introduce selection bias, which should be taken into account when interpreting the results.

### Questionnaire

Data on KAP related to osteoporosis prevention, along with lifestyle habits, were collected using structured questionnaires. The Osteoporosis Prevention and Awareness Test (OPAAT) was employed to evaluate knowledge levels (Toh et al., 2015; Ahmed et al., 2023). This instrument includes 30 questions covering topics such as the pathophysiology of osteoporosis, preventive strategies, and the consequences of untreated disease. Scores were assigned based on participant responses: 1 point for each correct answer and 0 points for incorrect answers or “do not know” responses. Knowledge levels were categorized as high (≥24/30), average (19–23), or low (<19).

In addition to knowledge assessment, sociodemographic and behavioral data were gathered. Variables included age, gender, body mass index (BMI), education level, residence, marital status, and the presence of comorbidities such as hypertension, diabetes, and heart disease. Lifestyle practices were also evaluated, distinguishing between positive behaviors (e.g., regular exercise, calcium and vitamin K2 consumption, sun exposure) and negative behaviors (e.g., smoking, alcohol use, and sedentary habits).

A self-administered questionnaire was distributed to patients upon admission in a paper-based format or electronically via WeChat, prior to receiving any education or information from their doctor. To ensure consistency, ward nurses received standardized training to guide patients in completing the survey. Incomplete responses were deemed invalid to maintain data integrity.

### Statistical analysis

Categorical variables were reported as frequencies and percentages. Chi-square tests were employed for categorical variables. A p-value of less than 0.05 was considered statistically significant. All statistical and descriptive analysis were performed by using SPSS 22.0 software (SPSS Inc., Chicago, IL, United States). A p-value <0.05 was considered statistically significant.

## Results

### Demographic characteristics

A total of 217 participants were included in the study. The majority were aged between 50 and 59 years (47%), followed by 60–69 years (40.6%), and ≥70 years (12.4%). Females accounted for 59.4% of the participants, while 40.6% were male. Most participants had a BMI within the range of 18.5–23.9 (54.8%), while 35.5% were classified as ≥24.0 kg/m^2^, and 9.7% had a BMI of <18.5. Educational levels varied, with 31.8% having a primary school education or less, 40.6% completing high school, and 27.5% attaining a college degree or higher. A larger proportion of participants resided in rural areas (58.5%) compared to urban areas (41.5%). The majority were married (79.7%). Regarding comorbidities, 43.3% had hypertension, 18.4% had diabetes, and 33.6% had heart disease (Table 1).

**TABLE 1:** Demographic data of 217 participants.

Variable	N (%)
Age	
50–59	102 (47%)
60–69	88 (40.6%)
≥70	27 (12.4%)
Gender	
Male	88 (40.6%)
Female	129 (59.4%)
Body mass index (kg/m2)	
<18.5	21 (9.7%)
18.5–23.9	119 (54.8%)
≥24.0	77 (35.5%)
Educational level	
Primary school or less	69 (31.8%)
High school	88 (40.6%)
College degree or high	60 (27.5%)
Residence	
Rural	127 (58.5%)
Urban	90 (41.5%)
Marital status	
Married	173 (79.7%)
Others	44 (20.3%)
Hypertension	
No	123 (56.7%)
Yes	94 (43.3%)
Diabetes	
No	177 (81.6%)
Yes	40 (18.4%)
Heart disease	
No	144 (66.4%)
Yes	73 (33.6%)
OPAAT score	
Low	70 (32.3%)
Average	85 (39.2%)
High	62 (28.6%)

### Knowledge, attitude, and awareness of osteoporosis prevention

Participants were categorized into low (32.3%), average (39.2%), and high (28.6%) knowledge levels based on their OPAAT scores (Table 1). Gender was significantly associated with knowledge levels (P = 0.012), with females more likely to have higher knowledge scores. Educational level also had a significant impact (P = 0.022); participants with a college degree or higher demonstrated higher knowledge. Rural residents were less knowledgeable compared to their urban counterparts (P = 0.009). Diabetes status was another significant factor (P = 0.011), as participants with diabetes tended to have higher knowledge scores (Table 2).

**TABLE 2:** Association of demographic characteristics with participants’ knowledge of osteoporosis prevention and awareness.

Variable	Low knowledge	Average knowledge	High knowledge	P Value
	n (%)	n (%)	n (%)	
Age				0.512
50–59	38 (37.3%)	40 (39.2%)	24 (23.5%)	
60–69	24 (27.3%)	35 (39.8%)	29 (33.3%)	
≥70	8 (29.6%)	10 (37.0%)	9 (33.3%)	
Gender				**0.012**
Male	37 (42.0%)	34 (38.6%)	17 (23.5%)	
Female	33 (25.6%)	51 (39.5%)	45 (34.9%)	
Body mass index (kg/m2)				0.971
<18.5	7 (33.3%)	8 (38.1%)	6 (28.6%)	
18.5–23.9	40 (33.6%)	47 (39.5%)	32 (26.9%)	
≥24.0	23 (29.9%)	30 (39.0%)	24 (31.2%)	
Educational level				**0.022**
Primary school or less	30 (43.5%)	26 (37.7%)	13 (18.8%)	
High school	29 (33.0%)	34 (38.6%)	25 (28.4%)	
College degree or high	11 (18.3%)	25 (41.7%)	24 (40.0%)	
Residence				**0.009**
Rural	50 (39.4%)	49 (38.6%)	28 (22.0%)	
Urban	20 (22.2%)	36 (40.0%)	34 (37.8%)	
Marital status				0.846
Married	57 (32.9%)	68 (39.3%)	48 (27.7%)	
Others	13 (29.5%)	17 (38.6%)	14 (31.8%)	
Hypertension				0.233
No	38 (30.9%)	54 (43.9%)	31 (25.2%)	
Yes	32 (34.0%)	31 (33.0%)	31 (33.0%)	
Diabetes				**0.011**
No	65 (36.7%)	66 (37.3%)	46 (26.0%)	
Yes	5 (12.5%)	19 (47.5%)	16 (40.0%)	
Heart disease				0.88
No	48 (33.3%)	56 (38.9%)	40 (27.8%)	
Yes	22 (30.1%)	29 (39.7%)	22 (30.1%)	

The statistical significance of P values is highlighted in bold.

### Healthy lifestyle behaviors

Regarding osteoporosis prevention practices, only 23% of participants engaged in outdoor exercise ≥3 times per week, 44.7% exercised 1–2 times weekly, and 32.3% reported no outdoor exercise. Calcium supplementation was reported by 64.5% of participants, while 41.0% used vitamin D supplements; however, only 10.6% included vitamin K2 in their regimen. Daily consumption of milk or dairy products was low, with only 26.7% reporting regular intake. Sun exposure habits revealed that 22.1% exposed their skin to sunlight ≥3 times weekly, 58.1% did so 1–2 times weekly, and 19.8% rarely had sun exposure. Smoking and alcohol consumption rates were relatively low, at 18.4% and 19.4%, respectively (Table 3).

**TABLE 3:** Distribution of some healthy life behavior characteristics of patients.

Variable	N (%)
Outdoor exercise	
≥3 per week	50 (23.0%)
1–2 times a week	97 (44.7%)
No	70 (32.3%)
Taking calcium supplements	
Yes	140 (64.5%)
No	77 (35.5%)
Taking a vitamin D supplements	
Yes	89 (41.0%)
No	128 (59.0%)
Taking vitamin K2 supplements	
Yes	23 (10.6%)
No	194 (89.4%)
Daily consumption of milk or dairy products	
Yes	58 (26.7%)
No	159 (73.3%)
How often should open parts of the body be in direct contact with the sun’s rays?	
≥3 per week	48 (22.1%)
1–2 times a week	126 (58.1%)
Hardly ever	43 (19.8%)
Smoking	
No	177 (81.6%)
Yes	40 (18.4%)
Alcohol	
No	175 (80.6%)
Yes	42 (19.4%)

## Discussion

The study revealed that gender, educational level, residence, and diabetes status significantly influenced osteoporosis prevention knowledge. Female participants demonstrated higher awareness, possibly due to greater health-related information-seeking behavior. Similarly, participants with college education or higher displayed better knowledge, underscoring the impact of educational attainment, which was in line with many previous studies (Tabor et al., 2022; Elgzar et al., 2023; Oumer et al., 2020). However, Alhouri et al. (2022) reported no significant association between educational level and osteoporosis knowledge, suggesting variability in results across different populations and contexts. Urban residents displayed higher levels of awareness compared to their rural counterparts, possibly due to better access to healthcare resources. This aligns with Alhouri et al. (2022), who also reported greater osteoporosis awareness among urban participants. Beyond healthcare access, additional factors may contribute to this disparity, including cultural perceptions of aging and bone health, lower literacy rates, and economic constraints (Oumer et al., 2020). Rural populations often have limited exposure to formal health education and may rely more on traditional beliefs, which can shape their health behaviors. To narrow this knowledge gap, targeted interventions such as community-based health programs, mobile health education initiatives, and expanded osteoporosis screening services in rural areas could be effective in improving awareness and preventive practices.

Notably, the study revealed a significant association between diabetes and increased osteoporosis awareness. A possible explanation is that individuals with diabetes have more frequent interactions with healthcare professionals for regular monitoring and treatment, which exposes them to more health education, including information on bone health. Moreover, diabetes management often incorporates lifestyle interventions—such as dietary modifications and physical activity—that overlap with osteoporosis prevention strategies. To address these disparities, targeted interventions, such as community health programs and educational initiatives in rural areas, should be implemented. These efforts could help bridge knowledge gaps and ensure equitable access to osteoporosis-related information and preventive resources. Further research exploring the role of chronic disease education in osteoporosis awareness could provide deeper insights into effective public health strategies.

The rates of calcium and vitamin D supplementation among participants were relatively high, at 64.5% and 41.0%, respectively. However, only 10.6% reported using vitamin K2 supplements, reflecting a limited understanding of comprehensive bone health practices. This aligns with findings in prior studies, which have highlighted the underutilization of vitamin K2 in osteoporosis prevention regimens, despite its role in regulating calcium metabolism and enhancing bone mineralization (Shiraki et al., 2010; Elahmer et al., 2024). The low prevalence of vitamin K2 supplementation in our study population may stem from a general lack of awareness, as most osteoporosis prevention efforts primarily emphasize calcium and vitamin D. Additionally, dietary sources of vitamin K2, such as fermented foods (Bonaldo et al., 2024), are not widely consumed in certain regions, further contributing to inadequate intake. To enhance awareness, targeted educational initiatives should emphasize the complementary role of vitamin K2 in bone health. Healthcare providers can integrate this information into routine patient counseling and postoperative care plans to ensure a more holistic approach to osteoporosis prevention. Furthermore, the low daily consumption of dairy products (26.7%) and inconsistent sun exposure observed in this study may result from factors such as limited dietary diversity, misconceptions about bone health, and lifestyle constraints. These behaviors could significantly affect vitamin D and calcium intake, highlighting the need for tailored interventions to promote healthier habits and improve osteoporosis prevention efforts.

Furthermore, only 23% of participants engaged in regular outdoor exercise, a crucial factor for maintaining bone density and overall health. This low participation rate aligns with findings from similar studies, such as a Korean cohort where approximately 80% of older adults aged 60–70 years did not engage in moderate physical activity, and inactivity increased to 90% in those over 71 years (Park et al., 2014). In contrast, Western populations often report slightly higher physical activity rates, attributed to better access to community fitness programs and culturally ingrained exercise habits (Zhang and Warner, 2023). These differences highlight the importance of infrastructure and cultural influences in promoting physical activity. To address the low participation in outdoor exercise, targeted interventions are needed. Healthcare providers can offer personalized counseling on the benefits of physical activity, while community-based and home exercise programs can provide accessible options for patients. Leveraging technology, such as mobile health apps and virtual classes, can further support engagement, especially for those with mobility limitations. Additionally, educational campaigns highlighting the role of weight-bearing exercises in osteoporosis prevention can help raise awareness and encourage behavior change. These strategies can bridge the gap and promote healthier lifestyles in this patient population.

Healthcare providers play a pivotal role in osteoporosis prevention. Integrating bone health education into routine postoperative care for lumbar fusion patients could enhance knowledge and attitudes. Additionally, implementing policy-level initiatives, such as subsidized calcium and vitamin supplements for at-risk populations, could improve accessibility. Leveraging technology, such as mobile health applications, can also facilitate the dissemination of targeted educational materials and monitor patient compliance with prevention strategies.

This study has several limitations. First, our study was conducted in a tertiary hospital, which may introduce some selection bias, as patients from different socioeconomic backgrounds and healthcare access levels may have varying knowledge and attitudes toward osteoporosis prevention. However, we aimed to include a diverse sample by recruiting both urban and rural residents and patients with different education levels. Future studies with multi-center data collection would help improve the generalizability of our findings. Second, the self-reported nature of the questionnaires may introduce response bias, as participants may overestimate positive behaviors. Lastly, the cross-sectional design precludes causal inferences about the relationships between demographic factors and knowledge levels. Future research should incorporate longitudinal follow-up studies to assess changes in knowledge, attitudes, and practices over time. Evaluating the effectiveness of targeted educational interventions on osteoporosis prevention behaviors would be a valuable extension of our current work.

## Conclusion

Older adult patients recovering from lumbar fusion surgery exhibit varied levels of knowledge, attitudes, and practices regarding osteoporosis prevention. Gender, education, residence, and comorbidities such as diabetes are significant determinants of awareness. While some positive behaviors, such as supplement use, were observed, gaps in exercise and dietary habits remain. This study emphasizes the importance of personalized educational interventions to improve bone health and prevent osteoporosis-related complications in this vulnerable group.

## Data Availability

The raw data supporting the conclusions of this article will be made available by the authors, without undue reservation.
